# ProtekDuo in Lung Transplant Candidates: Insights From a Single-Center Study of Cases

**DOI:** 10.1016/j.atssr.2025.09.011

**Published:** 2025-10-08

**Authors:** Domingo J. Franco-Palacios, Sharon Raymond-Forde, Kaitlin Olexsey, Lisa Allenspach, Vikas Aggarwal, Pedro Engel, Tiberio Frisoli, Austin Parsons, Jane Simanovski, Eric Simpson, Paul Villalba, Kyle Miletic

**Affiliations:** 1 Division of Pulmonary and Critical Care Medicine, Henry Ford Hospital, Detroit, Michigan; 2 Division of Cardiovascular Medicine, Henry Ford Hospital, Detroit Michigan; 3 Department of Internal Medicine, Henry Ford Hospital, Detroit, Michigan; 4 Heart and Vascular Institute, Henry Ford Hospital, Detroit, Michigan; 5 Department of Anesthesiology, Henry Ford Hospital, Detroit, Michigan; 6 Thoracic Surgery, Henry Ford Hospital, Detroit, Michigan

## Abstract

**Background:**

Right ventricular dysfunction often complicates severe lung disease or arises in patients on venovenous extracorporeal membrane oxygenation. Hemodynamic instability can disqualify patients awaiting lung transplant (LT). The ProtekDuo cannula, a dual-lumen device, offers simultaneous right ventricle support and oxygenation when connected to an in-line oxygenator.

**Methods:**

This single-center retrospective study reviewed patients bridged to LT with a ProtekDuo cannula between June 14, 2021, and January 25, 2024.

**Results:**

ProtekDuo was used in 9 patients, in 6 as a bridge to decision and in 3 as a bridge to transplant. It was the initial support in 5 patients; the rest transitioned from venovenous extracorporeal membrane oxygenation because of right ventricular failure (median, 11 days; interquartile range, 7.5-24 days). Of 7 patients listed for LT, 3 underwent LT; 1 patient was successfully bridged with ProtekDuo alone, whereas 2 patients required alternative support because of complications before LT.

**Conclusions:**

Matching patients with optimal mechanical support remains critical, especially given the high mortality of patients on extracorporeal life support on the LT waitlist. Although the ProtekDuo offers dual organ support, further investigation is needed to establish its role as a bridging strategy.

**In Short**

▪ We identified 2 potential situations in which the ProtekDuo cannula, used as an oxygenated right ventricular assist device, could effectively serve as a bridge to lung transplantation.
▪ One potential situation is worsening right ventricular dysfunction in patients on venovenous extracorporeal membrane oxygenation.
▪ Another potential situation is development of right ventricular failure not due to fixed pulmonary arteriopathy in patients with acute respiratory failure.

In 2016, Diaz-Guzman and coworkers^1^ described the case of a 43-year-old woman with scleroderma-related interstitial lung disease and pulmonary hypertension (PH) who had acute cor pulmonale and respiratory arrest treated with venoarterial (VA) extracorporeal membrane oxygenation (ECMO). Once the patient was stable, they reconfigured ECMO from VA to a CentriMag blood pump (Abbott Cardiovascular) connected to the ProtekDuo cannula, resulting in improved cardiac output and oxygenation that allowed ambulation. This patient underwent a successful lung transplant (LT) and represented the first case of ProtekDuo used as a bridge to LT (BTT).

Two systematic reviews and 2 single-center studies^2^, ^3^, ^4^, ^5^ have documented the use of various cannula strategies to support the right ventricle that when connected with an oxygenator offer both cardiac and pulmonary support. This configuration is referred to as an oxygenated right ventricular assist device, or OxyRVAD. It has been proposed as a strategt to delay or prevent escalation to VA ECMO in patients with respiratory failure and mild right ventricular (RV) dysfunction, offering respiratory and hemodynamic stability, sometimes as a BTT.

Capoccia and associates^2^ reported an intensive care unit survival rate of 63.9% and a LT success rate ranging from 71% to 100% among patients supported on OxyRVAD. Another review^3^ found OxyRVAD used as BTT or curative surgery in 37.4% of cases, with ProtekDuo specifically used in 12.

The ProtekDuo cannula (LivaNova PLC) is a 29F or 31F dual-lumen cannula originally designed for pulmonary support. When connected with an oxygenator in-line, it bypasses the right ventricle and provides venovenous (VV) ECMO, reducing right atrial pressure and RV preload while preserving transpulmonary blood flow and increasing pulmonary artery pressures and left ventricular (LV) preload. It is inserted percutaneously through the internal jugular vein and advanced into the pulmonary artery. The inflow lumen is positioned in the right atrium and the outflow lumen in the pulmonary artery. When used with an ECMO pump, flows of up to 4 L/min can be achieved. Its single-site cannulation avoids femoral access, minimizes recirculation (by placing the tricuspid and pulmonary valves between the drainage and return cannulas), and facilitates mobilization.^4^

## Patients and Methods

This single-center retrospective observational study examined LT candidates bridged to decision or transplant with a ProtekDuo cannula between June 14, 2021, and January 25, 2024. Approved by the institutional review board with a waiver of informed consent (protocol 17037; approved March 13, 2024), the study collected demographic and clinical data to evaluate mechanical circulatory support (MCS) strategies, outcomes, and complications related to the ProtekDuo or ECMO circuit.

## Results

During the study period, 227 patients underwent orthotopic LT at our hospital. ECMO was necessary in 23 patients as a bridge to evaluation and decision or as a BTT in those who were listed before cannulation. The main ECMO strategy was VV ECMO with a femoral-femoral cannulation or a double-lumen cannula using the Avalon (Getinge) or Crescent (Medtronic) cannula in the internal jugular or subclavian vein. ProtekDuo was used in 9 patients: as a bridge to evaluation and decision in 6 patients, and as a BTT in another 3 patients. The primary diagnosis was pulmonary fibrosis in 4 patients, acute respiratory distress syndrome in 3 patients, pulmonary arterial hypertension in 1 patient, and PH–chronic obstructive pulmonary disease in 1 patient. The baseline clinical characteristics are depicted in Table 1.

**Table 1:** Description of Our Cohort of Patients Who Remained Active in the Lung Transplant Waitlist

Patient	Underlying Diagnosis	ECMO Indication	MCS, Order	Complications
42-year-old man^a^	PCPF	BTD	ProtekDuo→V-AV ECMO	Small intracranial hemorrhage
63-year-old man	PH-COPD	BTT	ProtekDuo→VA ECMO	Chugging, cannula prolapsed in RV, air embolism, cardiac arrest
26-year-old woman^a^	Drug–acute respiratory distress syndrome	BTD	RIJ VV ECMO→ ProtekDuo→Femoral-femoral VV ECMO	Prolonged Protek Duo (49 days) with malfunction
65-year-old man	IPF	BTD	RIJ VV ECMO→ ProtekDuo	Gastrointestinal bleeding
58-year-old man	IPF	BTD	Femoral-femoral VV ECMO→ProtekDuo	RV failure with multiorgan failure
66-year-old woman^a^	IPF	BTT	ProtekDuo	Complete heart block during placement, severe TR
63-year-old woman	PH-LAM	BTT	ProtekDuo→VA ECMO	Worsening RV failure, biventricular failure

BTD, bridge to evaluation and decision; BTT, bridge to lung transplant; COPD, chronic obstructive pulmonary disease; ECMO, extracorporeal membrane oxygenation; IPF, idiopathic pulmonary fibrosis; LAM, lymphangioleiomyomatosis; MCS, mechanical circulatory support; PCPF, post–COVID-19 pulmonary fibrosis; PH, pulmonary hypertension; RIJ, right internal jugular vein; RV, right ventricle; TR, tricuspid regurgitation; VA, venoarterial; V-AV, venoarterial-venous; VV, venovenous.

a Patients who received lung transplant.

In our center, cannulations are performed by a cardiothoracic surgeon at the bedside on an urgent basis or by an interventional cardiologist in the catherization lab in more stable conditions. We use 2 different centrifugal pumps at our institution: the Abbott CentriMag pump and the Getinge Cardiohelp pump. For most patients, we use heparin infusion following a bolus of 5000 to 10,000 units at time of cannulation. Anticoagulation monitoring is based on measurement of anti-Xa level (target, 0.2-0.5 U/mL). If the patient is allergic to heparin or heparin-induced thrombocytopenia develops, the heparin infusion is switched to a direct thrombin inhibitor, mainly with bivalirudin. If bleeding develops, the ECMO circuit will be run without anticoagulation, provided the flows are higher than 2.5 L/min.

Different MCS strategies were used to support patients before LT, with frequent modifications in configuration and type of MCS based on changes in clinical condition of the patients. Median MCS duration was 11 (interquartile range, 7.5-24) days. VV ECMO with a double-lumen catheter in the right internal jugular vein and femoral-femoral VV ECMO were used in 4 patients as the initial MCS and before upgrading to ProtekDuo (Table 2). Reasons to change from VV ECMO to ProtekDuo were development of RV dysfunction in 2 patients, to facilitate mobilization in 1 patient with COVID-19–acute respiratory distress syndrome (CARDS) with femoral-femoral configuration, and worsening oxygenation in another patient. In 5 patients, ProtekDuo was the initial MCS strategy. The reasons the team chose the ProtekDuo in these patients were RV dysfunction in 4 patients and acute cor pulmonale secondary to CARDS in another patient. Of the 5 patients initially supported with ProtekDuo, 1 required VA ECMO during cardiopulmonary resuscitation, and 3 others with severe PH and RV dysfunction also needed MCS escalation to VA ECMO. Biventricular failure developed in 1 of them while on ProtekDuo (Table 2). The median duration on ProtekDuo was 7 (interquartile range, 4-29) days.

**Table 2:** ProtekDuo as the Initial MCS

Patient	Underlying Diagnosis	Echocardiogram Before ProtekDuo	RHC Before ProtekDuo (mm Hg)	MCS, Order	Outcome
42-year-old man	PCPF	Moderate RV dilation, moderate RV dysfunction, RVPO, eRVSP 62 mm Hg	RAP 16 PAP 80/32 (mean 50) PCWP 16 PA Sat 47%	ProtekDuo--> V-AV ECMO	LT
63-year-old man	PH-COPD	Severe RV dilation, severe RV dysfunction, RVPVO, eRVSP 75 mm Hg	RAP 16 PAP 76/28 (mean 52) PCWP 14 PA Sat 43%	ProtekDuo--> VA ECMO (eCPR)	Anoxic encephalopathy, died after withdrawal of MCS
66-year-old woman	IPF	Mild dilation of RV, normal RV systolic function, eRVSP 50 mm Hg	RAP 1 PAP 29/7 (mean 16) PCWP 9 PA Sat 71%	ProtekDuo	LT
45-year-old man	CARDS	Mild dilation of RV with mild systolic dysfunction, eRVSP 39 mm Hg	…	ProtekDuo	CARDS survivor
63-year-old woman	PH-LAM	Severe RV dilation, moderate RV dysfunction, RVPVO, eRVSP 78 mm Hg	RAP 11 PAP 111/44 (mean 76) PCWP 14 PA Sat 49%	ProtekDuo--> VA ECMO	Biventricular failure with multiorgan failure, died after withdrawal of MCS

CARDS, COVID-19–acute respiratory distress syndrome; COPD, chronic obstructive pulmonary disease; ECMO, extracorporeal membrane oxygenation; eCPR, ECMO cardiopulmonary resuscitation; eRVSP, estimated right ventricular systolic pressure by echocardiography; IPF, idiopathic pulmonary fibrosis; LAM, lymphangioleiomyomatosis; LT, lung transplant; MCS, mechanical circulatory support; PA Sat, pulmonary artery oxygen saturation; PAP, pulmonary artery pressure; PCPF, post–COVID-19 pulmonary fibrosis; PCWP, pulmonary capillary wedge pressure; PH, pulmonary hypertension; RAP, right atrial pressure, RHC, right-sided heart catheterization; RV, right ventricle; RVPO, right ventricle pressure overload; RVPVO, right ventricle pressure and volume overload; VA, venoarterial; V-AV, venoarterial-venous.

Ultimately, 7 of the 9 patients were listed for LT and 3 patients received a LT. Two patients with CARDS were removed from the LT list as they recovered and were discharged alive from the hospital without LT. Two patients were removed from the LT list because of being too ill to undergo LT and died after MCS withdrawal.

One patient was supported with ProtekDuo immediately before LT, whereas the other 2 patients who received transplants required a different MCS approach. One patient was converted to venoarterial-venous ECMO after 8 days on ProtekDuo because of hemodynamic instability with worsening PH during lightening of the sedation; and the other patient had a prolonged run on ProtekDuo for 49 days that led to blood clots and malfunction (converted to VV ECMO). Of the 3 patients who underwent transplantation, 2 patients received intraoperative VA ECMO, whereas 1 patient was supported with cardiopulmonary bypass. A 66-year-old woman with idiopathic pulmonary fibrosis who was bridged to LT with ProtekDuo died on hospitalization day 106 after LT. The other 2 patients who received transplants survived hospital discharge. Complications associated with the ProtekDuo were common (Table 1).

## Comment

From 2016 to December 2023, 2 systematic reviews along with data from single centers^6^, ^7^, ^8^, ^9^, ^10^, ^11^, ^12^ described the use of ProtekDuo for cardiopulmonary support before LT. Few of these studies discussed associated complications. Including this report, a total of 25 patients were identified in whom the ProtekDuo was used as OxyRVAD to bridge patients to LT.

Of these 25 patients, 76% (19/25) successfully underwent LT. The patients’ median age was 51 years (interquartile range, 43-55 years), 56% were male, and 48% had pulmonary fibrosis. We observed variability in the timing of and indication for ProtekDuo initiation in the reported cases. Some patients received VA ECMO for hemodynamic support, for example, in cases of cardiogenic shock due to RV failure, before ProtekDuo cannula placement.^1^^,^^6^ Switching from VA ECMO to the ProtekDuo not only facilitated earlier rehabilitation but also reduced complications associated with peripheral VA ECMO, such as device or cannula malfunction, access site complications, thromboembolism, myopathy, bleeding, and limb ischemia. Other patients with isolated respiratory failure and normal RV function were managed with VV ECMO and were later transitioned to Protek-Duo when RV dysfunction developed in a later stage or as a result of thrombosis or malfunctioning of the ProtekDuo.^9^^,^^12^

In some cases of end-stage lung disease with concurrent RV dysfunction, the ProtekDuo was used as the initial MCS strategy,^6^^,^^8^ which was the approach for 5 of our patients. In our cohort, although ProtekDuo was used to bridge patients to transplant, frequent changes in MCS configuration were made, and only 1 patient underwent LT while on the ProtekDuo. The same management was also seen in the case series described by Harano and coworkers,^8^ in which 2 of 4 patients on initial ProtekDuo VV ECMO were upgraded to venoarterial-venous ECMO and ultimately underwent LT.

When VV ECMO is employed as a MCS bridge, careful monitoring of RV function is recommended, as RV failure may develop during the course of support. Frequent clinical assessments, including evaluation of RV function with echocardiography and pulmonary artery catheterization for invasive hemodynamics,^13^ are crucial for guiding the cannulation strategy, selecting the type of MCS, or determining whether MCS upgrade is needed after a stepwise approach. We agree with the approach to MCS proposed by Beaulieu and coworkers,^3^ who recommended using the OxyRVAD for respiratory and RV support in the case of acute lung disease at higher risk of progression to RV failure (eg, acute respiratory distress syndrome), with no or only mild PH and isolated RV dysfunction. Conversely, severe lung disease with severe PH and RV dysfunction may require VA ECMO. In cases of new left-sided chamber distention, increasing LV filling pressure from concomitant LV dysfunction, a modified MCS that allows LV venting should be considered.^3^

Whereas ProtekDuo was successfully utilized as a BTT in over 70% of previously published cases, only 3 of 7 patients in our cohort underwent transplantation. Two patients required additional support with VA ECMO and venoarterial-venous ECMO. In these cases, severe PH associated RV dysfunction necessitated conversion to VA ECMO. When fixed pulmonary arteriopathy results in severe PH, it is possible that the ProtekDuo could cause more harm. In this case, the augmented transpulmonary flow will increase the pulmonary pressure, heightening the risk of pulmonary hemorrhage. Furthermore, it could worsen pulmonary vein regurgitation and may cause pulmonary edema by swiftly increasing the blood flow returning to a noncompliant left ventricle.

We observed several complications in this cohort of patients supported on the ProtekDuo. It is possible that publication bias exists, favoring reports mostly of successful bridges with the ProtekDuo, or the poorer outcome seen in our cohort is due to underlying comorbid conditions and severity of illness. As noted by several centers, some patients on the ProtekDuo as a BTT recovered without needing LT. This was seen particularly in patients with acute illness, as was the case with CARDS. Two of our listed patients with CARDS on the ProtekDuo survived their hospitalization without requiring an LT.

Our analysis of the reported patients’ characteristics does not provide sufficient information for definitive conclusions to be drawn about patient selection for this MCS modality. The 2022 expert consensus from The American Association for Thoracic Surgery suggests considering conversion to an OxyRVAD or full VA ECMO support for patients on VV ECMO who experience hemodynamic instability due to worsening RV function. This decision considers several factors, like urgency, relative risk of pulmonary hemorrhage, expected timing of transplantation, and anatomic limitations. The same guideline suggests that VA ECMO should be the MCS to BTT in patients with end-stage PH with RV failure.^14^

Adjusting the configuration of MCS is often necessary, highlighting the importance of understanding the benefits and risks of each device and the changes in clinical conditions. This could ensure the selection of the most suitable strategy for the fragile patient awaiting an LT.^13^^,^^14^

From the successful cases reviewed in this manuscript, we identified 2 possible scenarios in which OxyRVAD with use of the ProtekDuo cannula could benefit patients as BTT: in patients on VV ECMO who have worsening RV dysfunction; and in patients with acute respiratory failure at higher risk for development of RV failure by mechanisms other than fixed pulmonary arteriopathy. On the basis of this cohort, patients with severe PH are better suited for VA ECMO as initial MCS, and the use of the ProtekDuo should be avoided.
